# Spontaneous perirenal hemorrhage in systemic lupus erythematosus: a rare case report and literature review

**DOI:** 10.1186/s12882-021-02424-9

**Published:** 2021-06-09

**Authors:** Youlu Zhao, Xiaoyu Jia, Xiaoqiang Tong, Guochen Niu, Rui Wang, Lijun Liu, Fude Zhou

**Affiliations:** 1 Department of Nephrology, Peking University First Hospital; Institute of Nephrology, Peking University; Key Lab of Renal Disease, Ministry of Health of China; Key Laboratory of Chronic Kidney Disease Prevention and Treatment, Ministry of Education of China; Research Units of Diagnosis and Treatment of Immune-mediated Kidney Diseases, Chinese Academy of Medical Sciences, Beijing, 100034 China; 2 Department of Interventional Vascular Surgery, Peking University First Hospital, Beijing, 100034 China; 3 Department of Radiology, Peking University First Hospital, Beijing, 100034 China

**Keywords:** Systemic lupus erythematosus, Spontaneous kidney rupture, Perirenal hematoma, Acute kidney injury

## Abstract

**Background:**

Spontaneous perirenal hemorrhage is relatively uncommon but may be life-threatening. There are some challenges in early diagnosis due to the lack of specific presentations.

**Case presentation:**

We report a case of spontaneous perirenal hemorrhage in a newly diagnosed systemic lupus erythematosus patient who initially presented with non-specific flank pain. Weakness and unstable vital signs were noted on admission. Abdominal ultrasonography and computed tomography revealed a sizable perirenal hematoma over the left retroperitoneal cavity. Renal arteriography identified active extravasation of contrast media from a distant branch of the left renal artery, and selective embolization effectively obliterated the bleeding spot. After cessation of bleeding, the patient received intensive immunosuppressive therapy for acute kidney injury and encephalopathy due to lupus. Her mental status recovered successfully, and she was withdrawn from short-term hemodialysis.

**Conclusions:**

Spontaneous perirenal hemorrhage in the condition of systemic lupus erythematosus was a rare clinical entity with life-threatening potential. Early accurate diagnosis of spontaneous renal hemorrhage requires both detailed clinical examination and radiologic studies. Interventional embolization is essential and effective for both diagnosis and treatment. A high index of suspicion is necessary to avoid missing this potentially fatal syndrome, especially in patients with an increased risk of bleeding.

## Background

Spontaneous perirenal hemorrhage is a rare and potentially fatal condition of non-traumatic renal subcapsular and retroperitoneal bleeding [1]. Iatrogenic causes such as recent biopsy or surgical procedures need to be excluded.

Patients may present with the classic “Lenk’s triad” of acute flank or abdominal pain, palpable flank masses, usually non-specific, and ultimately fulminant hypovolemia. Computerized tomography (CT) and ultrasonography are the primary imaging options, and treatment varies from renal artery embolization to nephrectomy, depending on the severity. Early recognition and accurate diagnosis require both detailed clinical examinations and radiologic studies [2].

A meta-analysis summarized the most common reported etiology of spontaneous perirenal hemorrhage: benign or malignant neoplasm, predominantly angiomyolipoma, followed closely by renal cell carcinoma [3]. Vascular disease is the second most common cause of spontaneous perirenal hematoma, where polyarteritis nodosa (PAN) accounts for most occurrences [3]. Spontaneous perirenal hemorrhage associated with systemic lupus erythematosus (SLE) is rarely reported, and the underlying causes might be distinctive. Herein, we report a case of life-threatening spontaneous perirenal bleeding in a female patient with newly onset SLE.

## Case presentation

A 33-year-old Chinese female patient presented to our department with facial and lower limbs edema for over 1 month. Over 1 month before admission, her serum creatinine was 61 μmol/L (normal range 44-133). Ten days ago, her serum creatinine elevated (125 μmol/L), with 24-h urinary protein 3.36 g (normal range 0-0.15), and urinary red blood cell 30-40/high power field (HPF) (normal range 0-3), serum albumin 14 g/L (normal range 40-55). Complete blood count showed low levels of platelet (84 × 10^9^/L, normal range 125-350), white cell count (1.98 × 10^9^/L, normal range 3.5-9.5), and hemoglobin (100 g/L). Her antinuclear antibodies were 1:10000 (normal range1:< 100), C3 was 0.134 g/L (normal range 0.6-1.5), C4 was 0.044 g/L (normal range 0.12-0.36), anti-dsDNA antibody (ELISA) > 800 IU/ml (normal range < 100), anti-Smith antibody 82 (normal range < 25). She was then diagnosed with SLE [4]. Renal ultrasonography showed normal kidney size and cortical thickness with increased echogenicity in the renal parenchymal.

Four days ago, her serum albumin was 11.6 g/L, with 24-h urinary protein 1.28 g/300 ml, urinary red blood cell 0-2/HPF, serum creatinine 179 μmol/L. Her hemoglobin was 113 g/L and platelet 161 × 10^9^/L, which increased possibly due to extracellular dehydration from nephrotic syndrome. She had been on intravenous methylprednisolone of 40 mg due to renal and hematological involvement, subcutaneous injection of low molecular weight heparin at 3075AXaIU, and diuretics since. One day before admission, her hemoglobin was 65 g/L, platelet 79 × 10^9^/L, and her urine full of red blood cells under HPF. She had no other medical history to report. The next day she was transferred to our department through an ambulance.

**Fig. 1:**
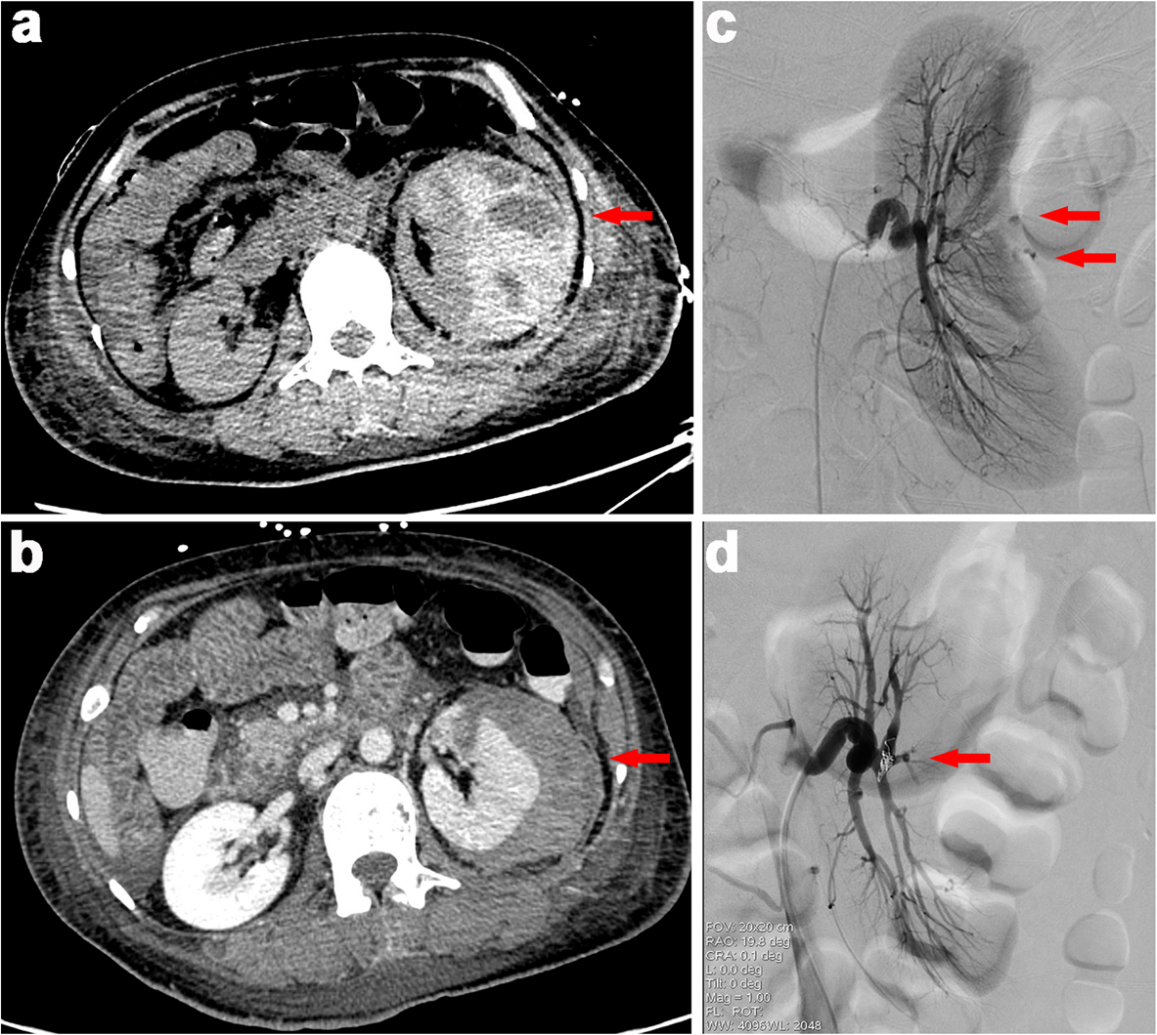
CT and renal artery angiography images: **a** Abdominopelvic non-contrast CT demonstrated abnormal left kidney contour that was interiorly displaced and externally compressed. A large but limited subcapsular collection with mixed density lateral to the left kidney was seen, with an overall size of 5.8 × 10.1 × 11.7 cm, red arrow; **b** Contrast-enhanced abdominopelvic CT showed renal parenchymal laceration> 1 cm in depth without collecting system rupture or urinary extravasation. The subcapsular hematoma was shrunk than before with an overall left kidney size of about 4.7 × 8.9 × 11.7 cm, red arrow. In contrast, the right kidney filled uniformly with intravenous dye and showed no obvious sign of injury; **c** The left kidney was significantly deformed due to suppression. Renal arteriography identified active contrast extravasation from a distant branch of the left renal artery, red arrow; **d** Post-embolization film showed successful obliteration of bleeding branch, red arrow

On admission, the patient was restless but responded correctly. Physical examination revealed pallor and relatively unstable vital signs (blood pressure, 149/85 mmHg; pulse, 130 beats/min, T 36.5 °C). Inspection revealed scattered ecchymosis due to anticoagulant injections on the abdomen. Abdominal palpation showed involuntary guarding and rigidity. Left flank tenderness on deep palpation was noted. Due to general weakness and frailty, it is hard for the patient to recall when the pain started creeping up. Initial investigations revealed a hemoglobin level of 42 g/L. Serum creatinine peaked at 296.2 μmol/L. Coagulation test was relatively normal except for high d-dimer level of 1.25 mg/L (normal range < 0.24 mg/L). Bedside abdominal ultrasonography showed a large left peri-renal fluid collection. There was no history of abdominal trauma. Therefore, spontaneous perirenal hemorrhage was highly suspected. A subsequent non-contrast abdominopelvic CT scan demonstrated subcapsular and perirenal hematoma of the left kidney (Fig. 1a). Emergency renal arteriography identified active extravasation of contrast media from a distant branch of the left renal artery (Fig. 1c). Furthermore, selective transcatheter embolization effectively obliterated the bleeding spot (Fig. 1d). With red blood cell transfusion and closely monitoring of hemoglobin, there was no sign of active bleeding. As the bleeding stopped, the patient continued 40 mg of methylprednisolone daily since admission for major organ involvement. Four days after admission, contrast-enhanced abdominopelvic CT confirmed renal parenchymal laceration (Fig. 1b).

Three days after admission, she manifested a sustained fever and cognitive defects. Her blood urea nitrogen was 63.99 mmol/L (normal range 1.8-7.1), and supportive hemodialysis was initiated in case of uremia encephalopathy. Except for lumbosacral decubitus ulcer infection, there were no signs of infection involving other systems. Neurologic examination reported no focal signs. Non-contract head CT was normal. Magnetic resonance imaging revealed slightly widened sulci and fissures of bilateral cerebral hemispheres. Since she could not cooperate, the cerebrospinal fluid test was postponed. Despite aggressive antibiotic therapy, headache and fever of over 38.5 °C persisted. As lupus encephalopathy was highly possible, she was administered three rounds of three-day intravenous pulses of methylprednisolone of 500 mg (since day six), followed by 40 mg methylprednisolone daily. She regained consciousness, and her fever resolved utterly after the pulse methylprednisolone. Renal replacement therapy was suspended after five times.

**Fig. 2:**
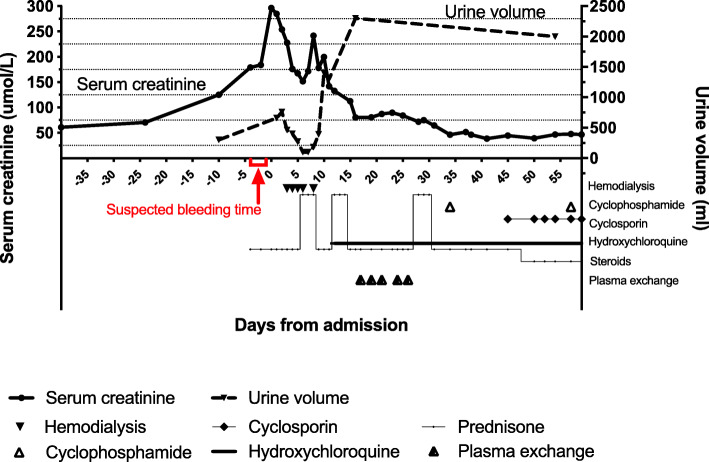
Time course of serum creatinine, urine volume, and relevant therapy

Due to her lumbosacral decubitus ulcer infection, a renal biopsy was unable to be performed. As she manifested as nephrotic syndrome with less prominent hematuria, lupus membranous nephropathy was highly suspected. After three pulses of methylprednisolone, she maintained 40 mg of methylprednisolone daily and two intravenous cyclophosphamide boluses (on day 34 and day 57 since admission, cumulative doses of 0.4 g). Her renal function gradually improved. One month after admission, her serum creatinine was restored to 46.3 μmol/, and urine volume increased to 2300 ml/d. Six weeks after admission, her albumin maintained a low level of 21 g/L, and cyclosporine 50 mg twice daily was added (Fig. 2).

**Fig. 3:**
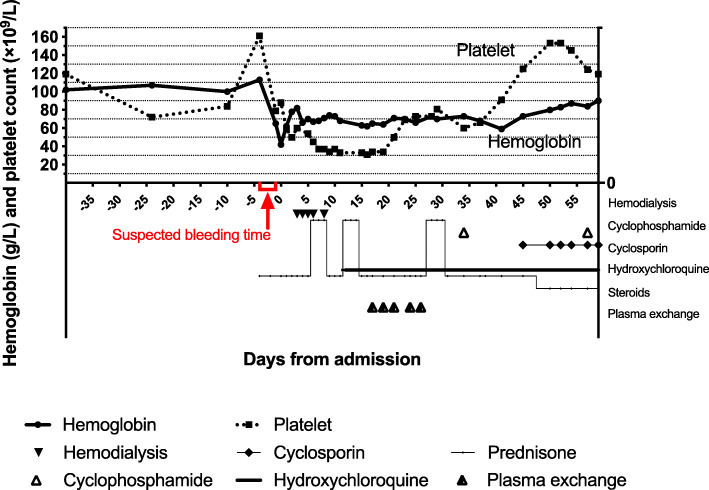
Time course of hemoglobin, platelet count, and relevant therapy

Thrombocytopenia had occurred since disease onset and did not improve after two rounds of intravenous pulses of methylprednisolone (Fig. 3). On day sixteen after admission, her platelet count reached the lowest level of 31 × 10^9^/L. Bone marrow aspiration smears showed normal thrombopoiesis. Antiplatelet antibody testing was negative. Lupus anticoagulant was absent. The anticardiolipin antibody of IgM and IgG isotype in serum was < 2.00 and < 12.0 PL-IgM-U/ml, respectively, within the normal range; and anti-β2-glycoprotein 1 antibody of IgG and IgM isotype in serum was 6.50 and 2.99RU/ml, respectively, within the normal range. Her lactate dehydrogenase was 956 IU/L (normal range 100-240), absolute reticulocyte percentage 6.15% (normal range 1.0-2.5), mean corpuscular volume (MCV) 83.4 femtoliters (normal range 82-100), international normalized ratio (INR) 0.84. Moreover, there were schistocytes observed on the peripheral blood smear. Her PLASMIC score was 6 points (hemolysis, no active cancer in the preceding year, no history of solid organ or hematopoietic stem cell transplant, MCV < 90 femtoliters, INR < 1.5, creatinine< 177 μmol/L**)**, indicating moderate/high risk for thrombotic microangiopathy (TMA). Plasma exchange was recommended under this condition. Meanwhile, waiting for ADAMTS13 results, the patient received five times plasma exchange. Later results reported that complement factor H, anti-factor H autoantibodies were within a normal range, and ADAMTS13 activity was 75%. Six weeks after admission, the patient’s platelet level finally reached 125 × 10^9^/L (Fig. 3).

The following recovery was uneventful, and 2 months after admission, she was discharged and with regular follow-up. Ten months after discharge, her prednisone was gradually tapered down to 5 mg daily, with cyclophosphamide 50 mg every other day fortnightly (cumulative dose of 6.8 g), and cyclosporin stopped 5 months after discharge. Her latest laboratory results were as follows: anti-dsDNA (ELISA) < 100 IU/ml, C3 0.875 g/L, C4 0.236 g/L, hemoglobin 109 g/L, platelet count 157 × 10^9^/L, serum creatinine 64 μmol/L, and albumin 37 g/L, urine protein +, urine red blood cell 0-1/HPF. Serologic measures and disease activity indices manifested her clinical remission.

## Discussion and conclusions

We report a female patient with severe manifestations of major organ involvement of SLE, and complicated by spontaneous rupture of the left kidney. With prompt diagnosis and treatment of transcatheter embolization for ruptured kidney, intensive intravenous methylprednisolone and supportive renal replacement therapy for active disease, her renal hematoma did not progress. Her renal function and mental status recovered successfully.

Spontaneous perirenal hemorrhage in the condition of SLE is relatively rare. A comprehensive search of articles from January 1980 through April 2020 was performed on Medline using the keywords perirenal hemorrhage, subcapsular or perinephric hematoma, systemic lupus erythematosus, and lupus nephritis. We excluded articles that referred to non-English, non-human studies, irrelevant or unpublished data. Potentially relevant studies were reviewed in full text. A total of 13 cases [5–17] were identified, and the underlying causes were detailed in Table 1. Among the reported cases, their age ranges between 21 and 58 years old. Female patients accounted for 75% (9/12). Three patients had SLE recently diagnosed, others with a history of SLE between 1.5-20 years.

**Table 1:** Underlying causes of SLE-associated spontaneous perirenal hemorrhage

No	Study	Age	Gender	SLE status^b^	SLE history (years)	Renal function	Proteinuria (g/d)	Vascular	Steroids	Immunosuppressant	Coagulopathy^a^	Cystic	Management	Outcome/follow-up (years)
1	Dux [5]	50	Female	Active	Recently	AKI,PD	2	Immune complex deposition	NA	NA	No	No	Nephrectomy	Alive/1
2	Si-Hoe [6]	NA	NA	NA	NA	NA	NA	Vasculitis	NA	NA	No	No	NA	NA
3	Mishriki [7]	36	Female	Inactive	12	Normal	No	No	Stop	AZA	No	No	Nephrectomy	Dead/0.3
4	Castellino [8]	46	Female	NA	15	NA	NA	No	No	HCQ	Yes	No	Conservative	Alive/discharge
5	Tsai [9]	21	Male	Active	Recently	Normal	2.1	Aneurysms	Increase	HCQ, AZA	No	No	Laparotomy	Alive/discharge
6	Chang [10]	30	Male	Inactive	6	HD	No	No	No	No	Yes	Yes	Conservative	Alive/discharge
7	Chen [16]	31	Female	Active	12	Normal	NA	Vasculitis	Increase	CTX	Yes	No	Conservative	Alive/discharge
8	Melamed [11]	36	Male	Active	10	Normal	Mild	PAN	Increase	CTX	No	No	Conservative	Dead/0.3
9	Chao [12]	39	Female	Active	20	Normal	Yes	Aneurysms	Continue	No	No	No	Embolization	Alive/discharge
10	Loureiro [13]	45	Female	Inactive	2	HD	No	No	No	No	Yes	Yes	Embolization	Alive/discharge
11	Ufuk [14]	30	Female	Active	7	HD	No	No	Continue	Methotrexate	Yes	No	Laparotomy	Alive/discharge
12	Bhusha [15]	30	Female	Active	Recently	Normal	2	PAN	Continue	Cellcept	No	No	Embolization	Alive/discharge
13	Wang [17]	58	Female	Active	1.5	CKD stage 2	3.2	No	Continue	CTX	Yes	No	Conservative	Alive/discharge
Our patient		33	Female	Active	Recently	AKI, HD	3.36	No	Increase	CTX, CsA, HCQ	Yes	No	Embolization	Alive/0.1

*Abbreviations*: *SLE* Systemic lupus erythematosus, *AKI* Acute kidney injury, *PD* Peritoneal dialysis, *HD* Hemodialysis, *PAN* Polyarteritis nodosa, *CTX* Cyclophosphamide, *CsA* Cyclosporin, *HCQ* Hydroxychloroquine, *AZA* Azathioprine, *NA* Not available. ^a^Denotes use of anticoagulants, thrombocytopenia, or antiphospholipid syndrome. ^b^Defined by SLE activity status descriptions in the paper or low C3, low C4 levels reported in the paper

Reviewing the literature, the most common causes for lupus-associated perirenal hemorrhage were vascular reasons, which accounted for 54% (7/13). Multiple microaneurysms consistent with PAN or renal artery aneurysm were observed under angiographic or imaging studies in four patients. There might be immune complex deposition on the vessel walls in active SLE-related vasculopathy [9, 12], which led to damages of vascular endothelium, arterial muscular, and elastic elements. Consequently, the vascular wall damage resulted in multiple small aneurysms in renal arteries within the kidney showed under radiographic [6, 9, 12], which subsequently resulting kidney rupture [18]. In the other three patients, though without radiographic confirmation, the authors attributed the bleeding to SLE-related vasculopathy. Dux et al. [5] described a spontaneous rupture of the kidney in a patient whose histologic findings were compatible with SLE. Nevertheless, there was no vascular aneurysm during the pathological examination; electron microscopic examination showed bulky subepithelial deposits causing thickening of the capillary wall, suggesting that the acute immune disorder might be the chief culprit.

About 58% (6/13) of patients with SLE had bleeding diathesis. In Castellino’s case [8], the patient had antiphospholipid syndrome and had been on warfarin for 6 months before pericapsular renal bleeding, and her INR at bleeding was as high as 10.4. In Chen’s case [16], the patient received aspirin and enoxaparin. In Wang’s case [17], the patient received heparin for nephrotic syndrome. The other three patients were receiving standard anticoagulant therapy due to maintenance hemodialysis. In the reviewed cases shown in Table 1, approximately 36% (4/11) patients were on renal replacement therapy, while 55% (6/11) of patients had a normal renal function, and 9% (1/11) patients experienced AKI. Fifteen% (2/13) of reported cases had cystic renal diseases [10, 13]. There were neither neoplasms nor infection (abscess or pyelonephritis) induced hemorrhage among the included cases.

In our case, several potential causes for renal bleeding required evaluation: immune-mediated vascular lesions, concomitant administration of anticoagulant, mild thrombocytopenia, and declined renal function might all be contributory. Firstly, the highly elevated anti-dsDNA antibody levels, decreased complement levels, severe lesions involving multisystem organ, and markedly central nervous system involvement suggested systemic lupus flare. Secondly, lupus microvascular lesions are relatively common, and their prevalence in SLE varied from 53.4 to 81.8% among different healthcare centers [19–21]. According to data from the University of Toronto Lupus Clinic, the existence of renal vascular lesions in 75.2% of the patients, in which arteriosclerosis was the most frequent lesion, occurring in 57.8% of patients, followed by TMA (8.1%) and vascular immune deposits (6.2%) [20]. The presence of TMA on renal biopsy was an independent risk factor for major bleeding in SLE patients [22]. In our patient, the presence of TMA was highly suspected, though she could not receive a renal biopsy to confirm renal microvascular lesions. Thirdly, a large amount of hematoma with high tension occupied the original place of normal renal vascular structure concealing the vascular changes. Though aneurysms were not seen during arteriography, other microvascular lesions could not be excluded. Besides, low molecular weight heparin before hemorrhage, TMA-induced thrombocytopenia, and acute declined renal function might also increase the bleeding diathesis and facilitate the event.

Disease activity of SLE varied among reviewed cases. Most (8/11) patients were reported to have either a high titer of anti-dsDNA antibody or low levels of C3 and C4, which were regarded as disease flares [23, 24]. Among those active cases, four patients reported having 24-h urinary protein ranging between 2 and 3 g. Patients with a large amount of proteinuria, hypoalbuminemia are at increased risk for thrombosis and bleeding after thrombosis. In Wang’s case [17], with lupus nephritis and nephrotic syndrome, the cause of retroperitoneal hematoma was left adrenal vein thrombosis resulting from the hypercoagulable state, which increased vascular pressure in the adrenal gland and eventually led to vascular rupture and hemorrhage. Given the clinical heterogeneity of SLE and the unpredictable disease course, steroids and immunosuppressive regimen is highly variable and is generally determined by disease activity [25]. Most patients (73%, 8/11) were under steroid therapy, 88% (7/8) received increased or continued doses of steroid therapy due to disease flares. Intensive immunosuppressive therapy could control lupus activity and immune-mediated vascular lesions [9, 11, 12, 14, 15]. In Tsai’s case [9], pulses of intravenous methylprednisolone 1000 mg were initiated, oral daily prednisone, azathioprine, and hydroxychloroquine were re-instituted under suspicion of vasculitis. In Melamed’s case [11], as bilateral multiple renal artery microaneurysms consistent with PAN diagnosis, the patient received methylprednisolone 1 g daily for three days followed by oral prednisone 60 mg daily and a pulse of intravenous cyclophosphamide 1000 mg. However, if the ruptured place was constituted of connective tissue, such as the junction between the renal pelvis and kidney [7], steroids were reduced or stopped to encourage healing due to the reason that glucocorticoids have a catabolic effect on connective tissue, muscle, fat, skin and wound healing [26]. In our case, the patient presented with high disease activity; after the resolution of the bleeding and restoration of hemodynamic stability, she was given intravenous pulses of methylprednisolone and had an uneventful recovery.

The management varies according to the hemorrhagic severity, overall clinical status, and other complications. 17% (2/12) of the reviewed patients underwent nephrectomy due to active bleeding. 42% (5/12) of the cases received conservative treatment, including blood transfusion, absolute bed rest, prophylactic antibiotic therapy, withdrawal from anticoagulant medications, and other supportive treatment. 15% (2/12) had explorative laparotomy due to unclear bleeding sources or secondary retroperitoneal infection. 25% (3/12) had transcatheter embolization to obliterate the hemorrhagic area. Most patients had uneventful recovery except for one patient [11] who died of post-pancreatic pseudocyst gastrotomy complications. The other patient died of disseminated intravascular coagulation with acute respiratory distress syndrome [7].

Spontaneous perirenal hemorrhage is relatively rare in SLE patients, and the etiologies varied. We report a female patient presented with spontaneous kidney rupture, together with severe manifestations of significant organ involvement of SLE. Prompt diagnosis of bedside ultrasound and abdominopelvic CT, treatment of selective coil embolization arrested the bleeding and stabilized the patient’s condition. Subsequent intensive immunosuppressive therapy controlled the disease and halted tissue injury. Her renal function, mental status, as well as hematological lesions recovered successfully.

## Data Availability

All data are presented in this manuscript.

## Abbreviations

CT: Computerized tomography
HPF: High power field
PAN: Polyarteritis nodosa
SLE: Systemic lupus erythematosus
MCV: Corpuscular volume
TMA: Thrombotic microangiopathy
ADAMTS13: A disintegrin and metalloprotease with a thrombospondin type 1 motif, member 13
